# (6,6′-Dimethyl-2,2′-bipyridine-κ^2^ *N*,*N*′)diiodidozinc(II)

**DOI:** 10.1107/S1600536809043049

**Published:** 2009-10-28

**Authors:** Robabeh Alizadeh, Khadijeh Kalateh, Zeinab Khoshtarkib, Roya Ahmadi, Vahid Amani

**Affiliations:** a Damghan University of Basic Sciences, School of Chemistry, Damghan, Iran; b Islamic Azad University, Shahr-e-Rey Branch, Tehran, Iran

## Abstract

The complete mol­ecule of the title compound, [ZnI_2_(C_12_H_12_N_2_)], is generated by crystallograpic twofold symmetry, with the Zn^II^ atom lying on the rotation axis. The Zn^II^ atom is coordinated by the *N*,*N*-bidentate 6,6′-dimethyl-2,2′-bipyridine ligand and two iodide ions, resulting in a distorted ZnN_2_I_2_ tetra­hedral geometry for the metal. In the crystal, there are weak π–π contacts between the pyridine rings [centroid–centroid distance = 3.978 (3) Å].

## Related literature

For related structures, see: Ahmadi *et al.* (2008 ▶, 2009 ▶); Alizadeh, Heidari *et al.* (2009 ▶); Alizadeh, Kalateh *et al.* (2009 ▶); Alizadeh, Khoshtarkib *et al.* (2009 ▶); Blake *et al.* (2007 ▶); Khalighi *et al.* (2008 ▶); Khan & Tuck (1984 ▶); Khavasi *et al.* (2008 ▶); Khoshtarkib *et al.* (2009 ▶); Kwak *et al.* (2008 ▶); Lee *et al.* (2007 ▶); Marjani *et al.* (2009 ▶); Reimann *et al.* (1966 ▶); Seebacher *et al.* (2004 ▶); Wriedt *et al.* (2008 ▶).
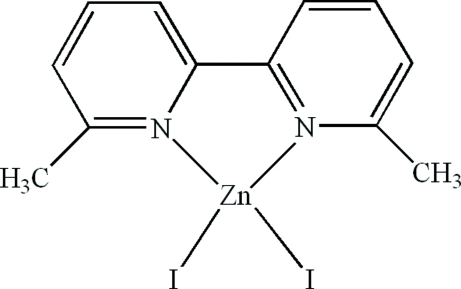


## Experimental

### 

#### Crystal data

[ZnI_2_(C_12_H_12_N_2_)]
*M* *_r_* = 503.43
Monoclinic, 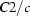

*a* = 13.421 (2) Å
*b* = 8.441 (2) Å
*c* = 13.752 (3) Å
β = 105.140 (14)°
*V* = 1503.8 (5) Å^3^
*Z* = 4
Mo *K*α radiation
μ = 5.72 mm^−1^
*T* = 298 K
0.48 × 0.12 × 0.11 mm

#### Data collection

Bruker SMART CCD diffractometer
Absorption correction: multi-scan (*SADABS*; Bruker, 1998 ▶) *T*_min_ = 0.425, *T*_max_ = 0.539
5694 measured reflections
1997 independent reflections
1748 reflections with *I* > 2σ(*I*)
*R*_int_ = 0.076

#### Refinement

*R*[*F* ^2^ > 2σ(*F* ^2^)] = 0.043
*wR*(*F* ^2^) = 0.127
*S* = 1.12
1997 reflections
79 parameters
H-atom parameters constrained
Δρ_max_ = 1.23 e Å^−3^
Δρ_min_ = −0.85 e Å^−3^

### 

Data collection: *SMART* (Bruker, 1998 ▶); cell refinement: *SAINT* (Bruker, 1998 ▶); data reduction: *SAINT*; program(s) used to solve structure: *SHELXS97* (Sheldrick, 2008 ▶); program(s) used to refine structure: *SHELXL97* (Sheldrick, 2008 ▶); molecular graphics: *ORTEP-3* (Farrugia, 1997 ▶); software used to prepare material for publication: *WinGX* (Farrugia, 1999 ▶).

## Supplementary Material

Crystal structure: contains datablocks I, global. DOI: 10.1107/S1600536809043049/hb5152sup1.cif

Structure factors: contains datablocks I. DOI: 10.1107/S1600536809043049/hb5152Isup2.hkl

Additional supplementary materials:  crystallographic information; 3D view; checkCIF report

## Figures and Tables

**Table:** 

Zn1—N1	2.058 (3)
Zn1—I1	2.5501 (6)

**Table:** 

N1—Zn1—N1^i^	81.9 (2)

Symmetry code: (i) 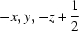.

**Fig. 1.**
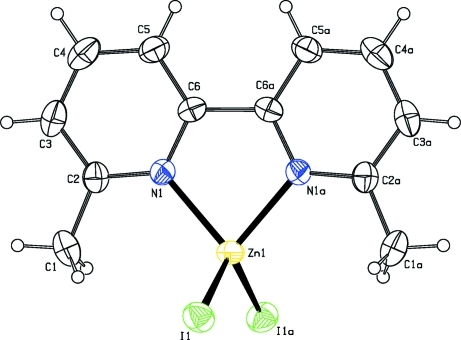
The molecular structure of (I) with displacement ellipsoids drawn at the 50% probability level.

**Fig. 2.**
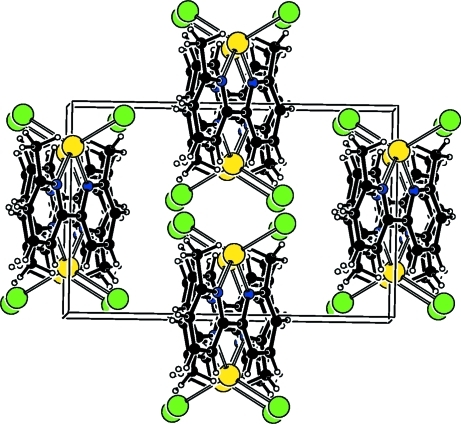
Unit-cell packing diagram for (I).

## supplementary crystallographic information

### Comment

Recently, we reported the synthes and crystal structure of [ZnCl_2_(phend)], (II), (Khoshtarkib *et al.*, 2009), [HgBr_2_(2,9-dmphen)], (III), (Alizadeh, Heidari *et al.*, 2009) and [Pb_4_(NO_3_)_8_(6-mbpy)_4_], (IV), (Ahmadi, Kalateh, Alizadeh *et al.*, 2009) [where phend is phenanthridine, 2,9-dmphen is 2,9-dimethyl-1,10-phenanthroline and 6-mbpy is 6-methyl-2,2'-bipyridine].

There are several Zn^II^ complexes, with formula, [Zn*X*_2_(N—N)], (*X* = Cl, Br and I), such as [ZnCl_2_(bipy)], (V), (Khan & Tuck, 1984), [ZnCl_2_(phen)], (VI), (Reimann *et al.*, 1966), [ZnCl_2_(dm4bt)], (VII), (Khavasi *et al.*, 2008), [ZnCl_2_(5,5'-dmbpy)], (VIII), (khalighi *et al.*, 2008), [ZnCl_2_(6-mbpy)], (IX), (Ahmadi, Kalateh, Ebadi *et al.*, 2008), [ZnCl_2_(6,6'-dmbpy)], (*X*), (Alizadeh, Kalateh *et al.*, 2009), [ZnCl_2_(PBD)]}, (XI), (Marjani *et al.*, 2009), [ZnBr_2_(4,4'-(dtbpy)].(Et_2_O), (XII), (Blake *et al.*, 2007), {ZnBr_2_[NH(py)_2_]},(XIII), (Lee *et al.*, 2007), {ZnBr_2_[S(py)_2_]}, (XIV) (Wriedt *et al.*, 2008), [ZnBr_2_(6,6'-dmbpy)], (XV), (Alizadeh, Khoshtarkib *et al.*, 2009), [ZnI_2_(2,9-dmphen)], (XVI), (Seebacher *et al.*, 2004) and {ZnI_2_[NH(py)_2_]}, (XVII) (Kwak *et al.*, 2008) [where bipy is 2,2'-bipyridine, phen is 1,10-phenanthroline, dm4bt is 2,2'-dimethyl-4,4'-bithiazole, 5,5'-dmbpy is 5,5'-dimethyl-2,2'-bipyridine, 6,6'-dmbpy is 6,6'-dimethyl-2,2'-bipyridine, PBD is *N*-(pyridin-2-ylmethylene)benzene-1,4-diamine, dtbpy is 4,4'-di-*tert*-butyl-2,2'-bipyridine, NH(py)_2_ is bis(2-pyridyl)amine, S(py)_2_ is bis(2-pyridyl)sulfide and NH(py)_2_ is bis(2-pyridyl)amine] have been synthesized and characterized by single-crystal X-ray diffraction methods. We report herein the synthesis and crystal structure of the title compound (I).

In the molecule of the title compound, (I), (Fig. 1), the Zn^II^ atom is four-coordinated in distorted tetrahedral configurations by two N atoms from one 6,6'-dimethyl-2,2'-bipyridine and two terminal I atoms. The Zn—I and Zn—I bond lengths and angles (Table 1) are within normal range (XVI).

The π-π contacts between the pyridine rings, *Cg*2···*Cg*2^i^ [symmetry cods: (i) –*X*,1-Y,1-*Z*, where, *Cg*2 is centroids of the ring (N1/C2—C6)] further stabilize the structure, with centroid-centroid distance of 3.978 (3) Å. It seems this π-π stacking is effective in the stabilization of the crystal structure (Fig. 2).

### Experimental

A solution of 6,6'-dimethyl-2,2'-bipyridine (0.20 g, 1.10 mmol) in methanol (10 ml) was added to a solution of ZnI_2_ (0.35 g, 1.10 mmol) in acetonitrile (10 ml) and the resulting colourless solution was stirred for 20 min at 313 K. This solution was left to evaporate slowly at room temperature. After one week, colourless needles of (I) were isolated (yield 0.41 g, 74.1%).

### Refinement

All H atoms were positioned geometrically, with C—H = 0.93–0.96Å and refined as riding with *U*_iso_(H) = 1.2*U*_eq_(C) or 1.5U_eq_(methyl C).

### Crystal data

**Table:**

[ZnI_2_(C_12_H_12_N_2_)]	*F*(000) = 936
*M**_r_* = 503.43	*D*_x_ = 2.224 Mg m^−^^3^
Monoclinic, *C*2/*c*	Mo *K*α radiation, λ = 0.71073 Å
Hall symbol: -C 2yc	Cell parameters from 896 reflections
*a* = 13.421 (2) Å	θ = 2.9–29.2°
*b* = 8.441 (2) Å	µ = 5.72 mm^−^^1^
*c* = 13.752 (3) Å	*T* = 298 K
β = 105.140 (14)°	Needle, colourless
*V* = 1503.8 (5) Å^3^	0.48 × 0.12 × 0.11 mm
*Z* = 4	

### Data collection

**Table:**

Bruker SMART CCD diffractometer	1997 independent reflections
Radiation source: fine-focus sealed tube	1748 reflections with *I* > 2σ(*I*)
graphite	*R*_int_ = 0.076
ω scans	θ_max_ = 29.2°, θ_min_ = 2.9°
Absorption correction: multi-scan (*SADABS*; Bruker, 1998)	*h* = −18→18
*T*_min_ = 0.425, *T*_max_ = 0.539	*k* = −9→11
5694 measured reflections	*l* = −18→18

### Refinement

**Table:**

Refinement on *F*^2^	Primary atom site location: structure-invariant direct methods
Least-squares matrix: full	Secondary atom site location: difference Fourier map
*R*[*F*^2^ > 2σ(*F*^2^)] = 0.043	Hydrogen site location: inferred from neighbouring sites
*wR*(*F*^2^) = 0.127	H-atom parameters constrained
*S* = 1.12	*w* = 1/[σ^2^(*F*_o_^2^) + (0.0623*P*)^2^ + 3.240*P*] where *P* = (*F*_o_^2^ + 2*F*_c_^2^)/3
1997 reflections	(Δ/σ)_max_ < 0.001
79 parameters	Δρ_max_ = 1.23 e Å^−^^3^
0 restraints	Δρ_min_ = −0.85 e Å^−^^3^

### Special details

**Table:**

Geometry. All e.s.d.'s (except the e.s.d. in the dihedral angle between two l.s. planes) are estimated using the full covariance matrix. The cell e.s.d.'s are taken into account individually in the estimation of e.s.d.'s in distances, angles and torsion angles; correlations between e.s.d.'s in cell parameters are only used when they are defined by crystal symmetry. An approximate (isotropic) treatment of cell e.s.d.'s is used for estimating e.s.d.'s involving l.s. planes.
Refinement. Refinement of *F*^2^ against ALL reflections. The weighted *R*-factor *wR* and goodness of fit *S* are based on *F*^2^, conventional *R*-factors *R* are based on *F*, with *F* set to zero for negative *F*^2^. The threshold expression of *F*^2^ > σ(*F*^2^) is used only for calculating *R*-factors(gt) *etc*. and is not relevant to the choice of reflections for refinement. *R*-factors based on *F*^2^ are statistically about twice as large as those based on *F*, and *R*- factors based on ALL data will be even larger.

### Fractional atomic coordinates and isotropic or equivalent isotropic displacement parameters (Å^2^)

**Table:**

	*x*	*y*	*z*	*U*_iso_*/*U*_eq_	
C1	0.1171 (5)	0.2312 (7)	0.4901 (4)	0.0650 (14)	
H1A	0.1495	0.1657	0.4501	0.078*	
H1B	0.0518	0.1858	0.4914	0.078*	
H1C	0.1607	0.2378	0.5575	0.078*	
C2	0.1003 (4)	0.3936 (6)	0.4452 (3)	0.0498 (9)	
C3	0.1376 (4)	0.5282 (8)	0.5028 (4)	0.0624 (13)	
H3	0.1737	0.5192	0.5702	0.075*	
C4	0.1190 (5)	0.6766 (8)	0.4564 (5)	0.0677 (14)	
H4	0.1435	0.7680	0.4925	0.081*	
C5	0.0640 (4)	0.6862 (6)	0.3567 (4)	0.0581 (11)	
H5	0.0499	0.7842	0.3251	0.070*	
C6	0.0304 (4)	0.5486 (5)	0.3045 (3)	0.0466 (9)	
N1	0.0498 (3)	0.4055 (4)	0.3488 (3)	0.0429 (7)	
Zn1	0.0000	0.22134 (8)	0.2500	0.0467 (2)	
I1	0.15441 (3)	0.07347 (4)	0.21872 (3)	0.06177 (17)	

### Atomic displacement parameters (Å^2^)

**Table:**

	*U*^11^	*U*^22^	*U*^33^	*U*^12^	*U*^13^	*U*^23^
C1	0.061 (3)	0.082 (4)	0.049 (3)	0.010 (3)	0.010 (2)	0.017 (2)
C2	0.045 (2)	0.062 (3)	0.043 (2)	0.0025 (19)	0.0125 (17)	−0.0009 (18)
C3	0.051 (2)	0.086 (4)	0.047 (2)	−0.001 (3)	0.0064 (19)	−0.017 (2)
C4	0.062 (3)	0.068 (3)	0.073 (3)	−0.009 (3)	0.017 (3)	−0.030 (3)
C5	0.058 (3)	0.046 (2)	0.068 (3)	−0.003 (2)	0.011 (2)	−0.011 (2)
C6	0.050 (2)	0.0395 (19)	0.052 (2)	−0.0028 (16)	0.0149 (18)	−0.0068 (16)
N1	0.0444 (17)	0.0428 (17)	0.0406 (16)	0.0000 (14)	0.0097 (13)	−0.0026 (13)
Zn1	0.0570 (4)	0.0354 (3)	0.0469 (4)	0.000	0.0121 (3)	0.000
I1	0.0680 (3)	0.0582 (2)	0.0608 (2)	0.01460 (14)	0.01977 (18)	−0.00003 (13)

### Geometric parameters (Å, °)

**Table:**

C1—C2	1.496 (8)	C4—H4	0.9300
C1—H1A	0.9600	C5—C6	1.378 (6)
C1—H1B	0.9600	C5—H5	0.9300
C1—H1C	0.9600	C6—N1	1.347 (6)
C2—N1	1.327 (6)	C6—C6^i^	1.508 (9)
C2—C3	1.400 (8)	Zn1—N1	2.058 (3)
C3—C4	1.398 (9)	Zn1—N1^i^	2.058 (3)
C3—H3	0.9300	Zn1—I1^i^	2.5501 (6)
C4—C5	1.379 (8)	Zn1—I1	2.5501 (6)
			
C2—C1—H1A	109.5	C6—C5—C4	119.0 (5)
C2—C1—H1B	109.5	C6—C5—H5	120.5
H1A—C1—H1B	109.5	C4—C5—H5	120.5
C2—C1—H1C	109.5	N1—C6—C5	121.5 (5)
H1A—C1—H1C	109.5	N1—C6—C6^i^	116.1 (2)
H1B—C1—H1C	109.5	C5—C6—C6^i^	122.4 (3)
N1—C2—C3	121.2 (5)	C2—N1—C6	120.5 (4)
N1—C2—C1	117.7 (4)	C2—N1—Zn1	126.6 (3)
C3—C2—C1	121.2 (5)	C6—N1—Zn1	112.8 (3)
C4—C3—C2	118.3 (5)	N1—Zn1—N1^i^	81.9 (2)
C4—C3—H3	120.8	N1—Zn1—I1^i^	113.38 (10)
C2—C3—H3	120.8	N1^i^—Zn1—I1^i^	110.04 (10)
C5—C4—C3	119.5 (5)	N1—Zn1—I1	110.04 (10)
C5—C4—H4	120.3	N1^i^—Zn1—I1	113.38 (10)
C3—C4—H4	120.3	I1^i^—Zn1—I1	121.39 (3)
			
N1—C2—C3—C4	0.9 (7)	C5—C6—N1—C2	1.6 (7)
C1—C2—C3—C4	−179.8 (5)	C6^i^—C6—N1—C2	−178.1 (5)
C2—C3—C4—C5	0.8 (8)	C5—C6—N1—Zn1	−175.7 (4)
C3—C4—C5—C6	−1.3 (8)	C6^i^—C6—N1—Zn1	4.7 (6)
C4—C5—C6—N1	0.1 (8)	C2—N1—Zn1—N1^i^	−178.8 (5)
C4—C5—C6—C6^i^	179.8 (6)	C6—N1—Zn1—N1^i^	−1.7 (2)
C3—C2—N1—C6	−2.1 (7)	C2—N1—Zn1—I1^i^	72.8 (4)
C1—C2—N1—C6	178.6 (4)	C6—N1—Zn1—I1^i^	−110.1 (3)
C3—C2—N1—Zn1	174.7 (3)	C2—N1—Zn1—I1	−66.8 (4)
C1—C2—N1—Zn1	−4.6 (6)	C6—N1—Zn1—I1	110.3 (3)

Symmetry codes: (i) −*x*, *y*, −*z*+1/2.

## References

[bb1] Ahmadi, R., Kalateh, K., Alizadeh, R., Khoshtarkib, Z. & Amani, V. (2009). *Acta Cryst.* E**65**, m848–m849. 10.1107/S1600536809024180 PMC2977193 21583318

[bb2] Ahmadi, R., Kalateh, K., Ebadi, A., Amani, V. & Khavasi, H. R. (2008). *Acta Cryst.* E**64**, m1266. 10.1107/S1600536808028894 PMC2959229 21201019

[bb3] Alizadeh, R., Heidari, A., Ahmadi, R. & Amani, V. (2009). *Acta Cryst.* E**65**, m483–m484. 10.1107/S1600536809009994 PMC2977550 21583736

[bb4] Alizadeh, R., Kalateh, K., Ebadi, A., Ahmadi, R. & Amani, V. (2009). *Acta Cryst.* E**65**, m1250. 10.1107/S1600536809038215 PMC2970226 21577766

[bb5] Alizadeh, R., Khoshtarkib, Z., Chegeni, K., Ebadi, A. & Amani, V. (2009). *Acta Cryst.* E**65**, m1311. 10.1107/S1600536809039610 PMC2971289 21578074

[bb6] Blake, A. J., Giunta, D., Shannon, J., Solinas, M., Walzer, F. & Woodward, S. (2007). *Collect. Czech. Chem. Commun.* **72**, 1107–1121.

[bb7] Bruker (1998). *SMART*, *SAINT* and *SADABS* Bruker AXS, Madison, Wisconsin, USA.

[bb8] Farrugia, L. J. (1997). *J. Appl. Cryst.* **30**, 565.

[bb9] Farrugia, L. J. (1999). *J. Appl. Cryst.* **32**, 837–838.

[bb10] Khalighi, A., Ahmadi, R., Amani, V. & Khavasi, H. R. (2008). *Acta Cryst.* E**64**, m1211–m1212. 10.1107/S1600536808027104 PMC2960607 21201646

[bb11] Khan, M. A. & Tuck, D. G. (1984). *Acta Cryst.* C**40**, 60–62.

[bb12] Khavasi, H. R., Abedi, A., Amani, V., Notash, B. & Safari, N. (2008). *Polyhedron*, **27**, 1848–1854.

[bb13] Khoshtarkib, Z., Ebadi, A., Alizadeh, R., Ahmadi, R. & Amani, V. (2009). *Acta Cryst.* E**65**, m739–m740. 10.1107/S160053680901959X PMC2969295 21582680

[bb14] Kwak, H., Lee, S. H., Kim, S. H., Lee, Y. M., Lee, E. Y., Park, B. K., Kim, E. Y., Kim, C., Kim, S. J. & Kim, Y. (2008). *Eur. J. Inorg. Chem.* pp. 408–415.

[bb15] Lee, Y. M., Hong, S. J., Kim, H. J., Lee, S. H., Kwak, H., Kim, C., Kim, S. J. & Kim, Y. (2007). *Inorg. Chem. Commun.* **10**, 287–291.

[bb16] Marjani, K., Asgarian, J., Mousavi, M. & Amani, V. (2009). *Z. Anorg. Allg. Chem.* **635**, 1633–1637.

[bb17] Reimann, C. W., Block, S. & Perloff, A. (1966). *Inorg. Chem.* **5**, 1185–1189.

[bb18] Seebacher, J., Ji, M. & Vahrenkamp, H. (2004). *Eur. J. Inorg. Chem.* pp. 409–417.

[bb19] Sheldrick, G. M. (2008). *Acta Cryst.* A**64**, 112–122. 10.1107/S0108767307043930 18156677

[bb20] Wriedt, M., Jess, I. & Näther, C. (2008). *Acta Cryst.* E**64**, m315. 10.1107/S1600536808000081 PMC2960344 21201286

